# Mitophagy: A New Player in Stem Cell Biology

**DOI:** 10.3390/biology9120481

**Published:** 2020-12-19

**Authors:** George Cairns, Madhavee Thumiah-Mootoo, Yan Burelle, Mireille Khacho

**Affiliations:** 1 Interdisciplinary School of Health Sciences, Faculty of Health Sciences, University of Ottawa, Ottawa, ON K1N 7K4, Canada; gcairns@uottawa.ca; 2 Department of Cellular & Molecular Medicine, Faculty of Medicine, University of Ottawa, Ottawa, ON K1H 8M5, Canada; mthum054@uottawa.ca; 3 Center for Neuromuscular Disease, Department of Biochemistry, Microbiology and Immunology, Faculty of Medicine, Ottawa Institute of Systems Biology (OISB), University of Ottawa, Ottawa, ON K1H 8M5, Canada

**Keywords:** mitophagy, stem cells, self-renewal, mitochondria, metabolism, mitochondrial quality control

## Abstract

**Simple Summary:**

Stem cells are required to create all organs and tissues during development, as well as replace or regenerate tissues in adulthood. Stem cells are characterized by two main factors, which are (1) the ability to replenish themselves in order to maintain their population for further use, and (2) the ability to differentiate into specialized cells. These two characteristics are regulated by both external and internal factors. One of these internal factors is mitochondrial function. Mitochondria are organelles that serve an essential role to cells by providing energy and regulating cell survival. These organelles are now known to be important for allowing the stem cell characteristics. Given that proper mitochondrial function is crucial for cells, when they become defective they need to be removed. This process of removal, known as mitophagy or “mitochondrial eating”, is emerging as an important player in stem cells. In this review we discuss the new research that shows the importance of mitophagy in having functional stem cells.

**Abstract:**

The fundamental importance of functional mitochondria in the survival of most eukaryotic cells, through regulation of bioenergetics, cell death, calcium dynamics and reactive oxygen species (ROS) generation, is undisputed. However, with new avenues of research in stem cell biology these organelles have now emerged as signaling entities, actively involved in many aspects of stem cell functions, including self-renewal, commitment and differentiation. With this recent knowledge, it becomes evident that regulatory pathways that would ensure the maintenance of mitochondria with state-specific characteristics and the selective removal of organelles with sub-optimal functions must play a pivotal role in stem cells. As such, mitophagy, as an essential mitochondrial quality control mechanism, is beginning to gain appreciation within the stem cell field. Here we review and discuss recent advances in our knowledge pertaining to the roles of mitophagy in stem cell functions and the potential contributions of this specific quality control process on to the progression of aging and diseases.

## 1. Introduction

Mitochondria are essential organelles within the cell responsible for a number of diverse functions including, energy metabolism, reactive oxygen species (ROS), Ca^2+^ dynamics, iron-sulphur cluster biogenesis and apoptosis. Given the involvement of mitochondria in such fundamental cellular pathways it is imperative that these organelles are maintained in an optimal state. As such, cells possess several quality control mechanisms to ensure the existence of functional mitochondria. Mitochondrial quality control (MQC) is an important process that encompasses three main pathways: the mitochondrial protease and chaperone system, the mitochondrial derived vesicle (MDV) pathway and mitophagy. Of these, mitophagy has emerged as an important mechanism not only in situations of mitochondrial dysfunction and during stress conditions, but also during physiological conditions of development and tissue regeneration. In this review we discuss recent developments highlighting novel roles for mitophagy in stem cells and how alterations in this pathway may be involved in stem cell dysfunctions during aging and disease states.

## 2. Mitophagy

**Figure 1:**
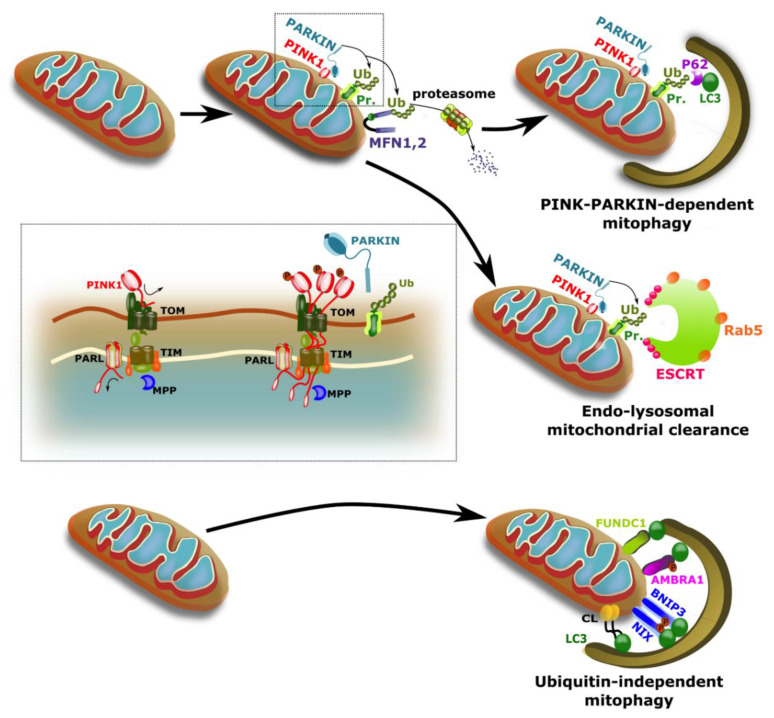
Overview of mitophagy pathways. PINK1/PARKIN-dependent mitophagy: Upon loss of membrane potential, PINK1 accumulates at the surface of mitochondria where it recruits PARKIN. Recruitment of this E3 ligase leads to the ubiquitination of several mitochondrial proteins, which in turn promote proteasomal degradation and recruitment of the core autophagy machinery, including P62 and LC3. Endo-lysosomal clearance: PARKIN-dependent ubiquitination of target proteins also leads to the recruitment of RAB5-positive early endosomes, which engulf mitochondria through a mechanism involving the ESCRT machinery. Ubiquitin independent mitophagy: Several proteins that are integral (FUNDC1, FKBP8) or are recruited to the outer mitochondrial membrane (BNIP3, NIX, AMBRA1, OPTINEURIN) contain LC3 binding sites that allow direct interaction with nascent autophagosomes under conditions of stress. Similarly, the phospholipid cardiolipin, can translocate from the inner to the outer mitochondrial membrane in response to mitochondrial dysfunction and directly bind the N-terminal region of LC3. (CL, cardiolipin; Ub, ubiquitin, Ub Pr., ubiquitinated protein).

Mitophagy is a selective form of autophagy in which mitochondria are specifically degraded. Similar to general autophagy (also called macro-autophagy), this process utilizes autophagosomes for the delivery and degradation of cargo in acidic lysosomes [1]. However, in contrast to general autophagy, in which autophagosomes contain bulk cellular materials (which can include mitochondria), mitophagy is characterized by the presence of autophagosomes containing solely mitochondria [2,3]. Mitophagy is thus specific to mitochondria, enabling dissociation from general autophagy and selective elimination of organelles in response to physiological cues or mitochondrial dysfunction/damage. Over the recent years, studies have led to the discovery of multiple mechanisms conferring specificity to the mitophagy pathway (Figure 1). However, the degree of overlap and the importance of each mechanism is yet to be fully understood.

### 2.1. Important Players in Mitophagy

*PINK1/PARKIN*: The most well studied pathway of mitophagy involves the mitochondrial serine/threonine PTEN-induced kinase 1 (PINK1) and the cytosolic E3-ligase PARKIN, two proteins found to be mutated in familial forms of early onset Parkinson’s disease [4,5]. Under basal conditions PINK1 is imported into mitochondria through the outer mitochondrial membrane (OMM) translocase of the outer mitochondrial membrane (TOM) complex and the translocase of the mitochondrial inner membrane (TIM23) complex located in the inner mitochondrial membrane (IMM) [6,7]. Following import, PINK1 is cleaved by the mitochondrial protease presenilin-associated rhomboid-like protein (PARL) in the IMM [8], resulting in two fragments (Figure 1). The PINK1 fragment residing in the mitochondrial matrix is degraded by the mitochondrial processing peptidase (MPP), and the m-AAA, while the remaining PINK1 is retro-translocated and degraded by the ubiquitin-proteasome system [7,9,10]. This rapid and continuous degradation of PINK1 protein under basal conditions results in very low steady-state levels that are barely detectable [7,9,10]. Import arrest of PINK1 was recently shown to rely on regulatory interactions between the adenine nucleotide translocator (ANT) and the TIM23-TIM44. The accumulation of PINK1 at the OMM promotes its dimerization and autophosphorylation, this allows the recruitment and phosphorylation of the E3 ubiquitin ligase PARKIN, which are both required for the efficient activation of PINK1/PARKIN-mediated mitophagy. Once ubiquitinated, surface proteins on mitochondria act as baits for the recruitment of the core autophagy machinery. P62/SQSTM1 was initially thought to act as an obligatory factor required for this recruitment phase [11]. However, more recent studies suggest that NDP52, OPTINEURIN and TAX1BP1 are key regulators of this process [12,13,14], while P62/SQSTM1 may be important for clustering of depolarized mitochondria but dispensable for the recruitment step [15]. Once present at the mitochondrial surface, these autophagy adaptor proteins bind the ATG8 family proteins (LC3/GABARAP) to initiate autophagosome formation [14]. At this step, studies suggest that the GTPases TBC1D15 and TBC1D17, which interact with the mitochondrial fission 1 protein (FIS1) and the endo-lysosomal trafficking protein RAB7, are required for proper encapsulation of mitochondria in autophagosomes [16,17].

In addition to autophagy-mediated mechanisms, the endo-lysosomal pathway was recently suggested to participate in the clearance of whole mitochondria [18]. One study has shown that following uncoupling using the mitochondrial uncoupler FCCP, RAB-5 positive early-endosomes are recruited to mitochondria along with the PIP3-producing VPS34 PIK3-BECLIN1 complex. Mitochondria are then internalized into endosomal membranes by the Endosomal Sorting Complexes Required for Transport (ESCRT) machinery (Figure 1), which are responsible for the invagination and subsequent scission of the endosomal membrane. Following maturation into RAB9-positive late endosomes, mitochondria are then delivered to lysosomes for degradation. Interestingly, the recruitment of the ESCRT machinery to mitochondria seems to depend on the ubiquitination of surface proteins by PARKIN (Figure 1), indicating a degree of overlap between the autophagic and the endo-lysosomal pathway of mitochondrial clearance [18].

Mitophagy can also proceed through several mechanisms that do not require ubiquitination and instead rely on the direct binding of LC3, present on nascent phagophores, to mitochondrial proteins and lipids. These mechanisms mainly involve (1) BNIP3L-NIX and BNIP3, (2) FUNDC1 (3) AMBRA1 and 4) cardiolipin (Figure 1), although a role for PROHIBITINs has recently been suggested.

*BNIP3L-NIX and BNIP3*: BNIP3L-NIX is closely related to the BCL2 family well known for their roles in apoptosis. However, early work on reticulocytes showed that BNIP3L-NIX plays an important role in the in the large-scale removal of mitochondria observed during differentiation into mature red blood cells [19,20]. (Figure 1). Although the full mechanism by which BNIP3L-NIX acts is not fully understood, it appears that dimerization in the OMM is important and that phosphorylation of BNIP3L-NIX on serine 34/35 leads to improved mitophagy [21,22]. Interestingly, the BCL2/adenovirus E1B 19kDa protein-interacting protein 3 (BNIP3) also plays a role in mitophagy. Similar to BNIP3L-NIX, this protein also harbors a LC3/GABARAP interaction domain, which allow direct recruitment of nascent autophagosomes to mitochondria [23]. Studies suggest that BNIP3 may be involved in hypoxia-induced mitophagy as BNIP3 is transcriptionally regulated by hypoxia-inducible factor 1a (HIFα-1a) [24].

*FUNDC1*: FUN14 Domain Containing 1 (FUNDC1) is also involved in hypoxia mediated mitophagy. Like BNIP3 and NIX, FUNDC1 is an OMM-localized protein that contains an LC3/GABARAP-binding motif which allows ubiquitin-independent recruitment of autophagosomes to mitochondria [25]. Recently FUNDC1 was reported to interact with DNM1L/DRP1 and OPA1 to help coordinate mitochondrial fission with hypoxia-induced mitophagy [26].

AMBRA1: Autophagy and Beclin 1 Regulator 1 (AMBRA1) has a well-known role in the regulation of general autophagy, where it has been described as a positive regulator of the BECLIN 1-dependent autophagy [27]. However, recent evidence suggests that AMBRA1 can also localize to the OMM where it is able to improve mitophagic clearance of depolarized mitochondria (Figure 1) [28]. Similar to other autophagy receptors present on the OMM, AMBRA1 contains a LC3-interacting region (LIR) motif enabling direct interaction with LC3/GABARAP on nascent autophagosomes [28]. Recent data indicate that the ability of AMBRA1 to interact with LC3/GABARAP is modulated through phosphorylation by HUWE1 and IKKa, which results in conformational changes in AMBRA1 [29]. In addition to this role in receptor-dependent mitophagy, AMBRA1 can also promote adaptor-mediated PINK1/Parkin-dependent mitophagy [28,30].

*Cardiolipin*: Cardiolipin, is a signature phospholipid of mitochondria generally found in the IMM. However, under stressed conditions cardiolipin can be found in the OMM where it interacts with LC3 (Figure 1). LC3 has cardiolipin binding domains that can facilitate autophagosome binding to mitochondria. Knockdown studies showed that cardiolipin is transported from the IMM by cardiolipin synthase or phospholipid scramblase-3 when mitochondria are exposed to a variety of stressors that inhibit the respiratory chain or stimulate ROS production and apoptosis such as rotenone, 6-hydroxydopamine and staurosporine [31].

PROHIBITIN 2: PROHIBITINs (PHB1 and PHB2) are localized to the IMM where they form large hetero oligomeric structures that act as protein and lipid scaffolds ensuring the integrity and functionality of the IMM [32]. Beyond these roles, recent data suggest that PROHIBITINs, particularly PHB2 can act as a mitophagy receptor [33]. Similar to OMM mitophagy receptors, PHB2 contains an LC3-interaction domain enabling direct binding to autophagosomal membranes upon mitochondrial membrane depolarization and rupture of the OMM [33,34], which can occur following proteasome-dependent degradation of OMM proteins or organelle swelling. While being able to bind LC3 directly, PHB2 also appears to be involved in PARKIN-dependent mitophagy through mechanisms that remain unclear. It is suggested that ubiquitin-proteasome-dependent rupture of the OMM may be involved in PARKIN-dependent mitophagy, which would allow the autophagic machinery direct access to PROHIBITIN complexes in the IMM [34].

### 2.2. Overlap and Cross Talk between Mitophagy Pathways

While a number of mitophagy players have been discovered, the overall picture is more complicated given the considerable degree of overlap between these pathways. For example, NIX was reported to contribute to PARKIN-mediated mitophagy by controlling mitochondrial translocation [35]. This has also been described with BNIP3, which can interact with PINK1 to halt its proteolytic cleavage and thus enhance mitophagy [36]. NIX is also able to compensate, at least partially, for the loss of PARKIN in both HeLa cells [37] and patient cells [38]. PINK1 has also been observed to induce mitophagy independently of PARKIN, whereby NDP52 and OPTINEURIN can directly recruit the autophagy machinery [39]. This can also be observed in animal models with a degree of compensation observed in in germline knockouts of PINK1 and PARKIN. In both models, only mild mitochondrial dysfunctions are present in tissues and loss of dopaminergic neurons, the hallmark of Parkinson’s disease, is minimal and only occurs in aged mice suggesting the existence of developmental compensation [40,41,42]. In line with this hypothesis, inducible knockout of PARKIN in the adult brain precipitates the loss of dopaminergic neurons [43], while deletion from the heart in neonates, blocks metabolic maturation in cardiomyocytes and precipitates dilated cardiomyopathy [44]. The degree of compensation from other pathways and cross talk between these pathways is not yet fully understood and more complex than initially considered.

## 3. Mitochondria and Stem Cells

Stem cells are the precursors for tissue development and regeneration throughout the lifetime of an organism. These cells have the unique ability to either self-renew or differentiate into various lineages [45,46]. The balanced proportion of these fate decisions is required to maintain homeostasis between the (re)generation of terminally differentiated cells, and the preservation of quiescent stem cell pools, which ensures the long-term maintenance of potency [46]. Maintenance of stemness and commitment to specific cellular lineages is regulated by environmental changes in stem cell niches, as well as cell-autonomous factors governing the expression of specific genetic programs [47]. For this reason, phenotypic heterogeneity exists within a given pool of stem cells with regard to stemness, with only a proportion representing the *bona fide* quiescent stem cells insuring long-term maintenance of potency [48,49,50]. Over recent years, mitochondria have emerged as important players not only in the maintenance of stem cell identify, but also for proper commitment and differentiation [46]. Although much remains to be learned, the emerging view is that transition from quiescence to commitment is linked to changes in state-defining mitochondrial properties. This section provides a brief overview of the mitochondrial properties generally associated with stemness, and the mitochondrial phenotype shifts associated with commitment and differentiation.

### 3.1. Mitochondrial Properties Associated with Stemness

One of the common characteristics of stem cells is the ability to maintain a low metabolic rate. This property is viewed as a conserved mechanism to limit wear and tear, and ensure long-term maintenance of potency. Consistent with this low energy need, most stem cells, including hematopoietic (HSC), embryonic (ESC) and mesenchymal (MSC) stem cells harbor a relatively small number of mitochondria with underdeveloped cristae [51,52,53]. Furthermore, although mitochondria can appear as rounded or more elongated depending on the type of stem cell, they generally form low complexity networks with only a few branch points, consistent with the low bioenergetic needs of quiescence [51,54,55,56,57]. In fact, a recent study analyzing HSC heterogeneity supports the existence of a strong link between restricted oxidative metabolism and maintenance of potency [58]. In this report, quiescent immunophenotypically defined HSCs were shown to maintain low mitochondrial activity based on mitochondrial membrane potential (MMP) and oxygen consumption rates. In contrast, cycling-primed HSC with lower stemness properties displayed increased MMP and oxygen consumption as well as higher glycolytic rates, consistent with cellular activation. While the necessity for restricting oxidative metabolism in stem cells is not fully understood, one of the obvious advantages is to limit the generation of reactive oxygen species (ROS) produced by multiple reactions within mitochondria including oxidative phosphorylation (OXPHOS) complexes and several metabolic enzymes (OGDH, PDH, BCKDH) [59]. This repression serves not only as a protective mechanism against oxidative damage but also as an effective brake of ROS signaling which plays a crucial role in stem cell fate decisions [51,52,54,60]. Low ROS levels are indeed known to preserve quiescence and self-renewing capacity, while increased ROS production is reported to act as a signaling mechanism driving proliferation and differentiation [51,52,54,60].

Although glycolytic metabolism, rather than OXPHOS, is reported to be the predominant source of energy in quiescent stem cells [61], recent data suggest that mitochondrial intermediary metabolism and OXPHOS, albeit limited, is nevertheless important for the maintenance of stemness. For instance, fatty acid metabolism driven by mitochondrial bioenergetics and mitochondrial network dynamics is reported to be important for maintenance of the self-renewal trait of stem cells including neural stem cells (NSC) and HSCs [62,63]. As a result, alteration of mitochondrial fatty acid oxidation (FAO) or mitochondrial dynamics cause an imbalance in stem cell fate decisions, leading to increased commitment of stem cells to a specific lineage at the expense of a decline in the stem cell pool [64,65]. Furthermore, disruptions to mitochondrial function, through pharmacological or genetic means, is known to result in severe stem cell dysfunctions, while improving mitochondrial function allows to maintain stemness in many stem cell populations [52,66].

**Figure 2:**
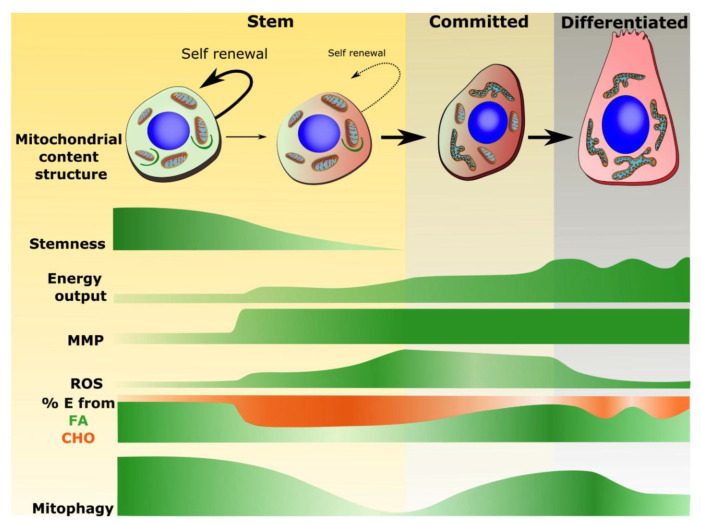
Integrative view illustrating the plasticity of the mitochondrial, metabolic and mitophagy landscape during stem cell lineage progression. Stem cells, although reliant on oxidative phosphorylation (OXPHOS) and fatty acid oxidation (FAO), display low energy outputs in the quiescent state. Hence, mitochondrial content, network complexity, and number of cristae folds is reduced. This low metabolic state is associated with a low mitochondrial electrochemical gradient, often equated to low Mitochondrial Membrane Potential (MMP), and reduced release of Reactive Oxygen Species (ROS). Heterogeneity exists within a stem cell population, with only a fraction presenting high stemness properties [58,69,70]. This latter population, representing the bona fide stem cells ensuring long-term maintenance of potency, present comparatively low rates of cell division and several features of metabolic stemness including low MMP, low ROS emission and increased reliance of FAO [53,58]. In contrast, stem cells harboring more active mitochondria, as evidenced by elevated MMP, increased oxygen consumption, and de-repression of OXPHOS from pyruvate (e.g., carbohydrates) present lower stemness properties, and are more prone to commit in response to activation signals [58]. Increased mitochondrial ROS, and potentially other metabolic cues, are suggested to act as signaling factors involved in the commitment of stem cells [53,68]. Once committed stem cells undergo highly orchestrated and lineage-specific mitochondrial remodeling programs which are required for normal differentiation. For instance, progenitors destined to differentiate into cells with high and/or sustained energy requirements, remodeling is generally characterized by increased mitochondrial biogenesis and network complexity, qualitative changes in mitochondrial ultrastructure, enhanced OXPHOS capacity, and heightened antioxidant capacity. Mitophagy is believed to be active in quiescent stem cells particularly those displaying the low MMP, low metabolic state [58], suggesting a role in the maintenance of metabolic stemness. Upregulation of mitophagy is also required during cellular differentiation which involves large-scale remodeling of mitochondria, particularly in energy demanding tissues [44]. Furthermore, evidence suggest that downregulation of mitophagy in stem cells promotes commitment. (E, energy; FA, fatty acid; CHO, carbohydrate).

Put together, these recent advances present a set of mitochondrial characteristics that define “*metabolic stemness*”, which are summarized in Figure 2. These include, but may not be limited to, low mitochondrial abundance, underdeveloped ultrastructure, low network complexity, restricted rates of OXPHOS with a relative reliance on fatty acid oxidation, reduced membrane potential, and low ROS emission. Beyond low ROS signaling, this specific mitochondrial profile likely contributes to the maintenance of stem cell-specific genetic programs through additional mechanisms, including modulation of important second messengers such as intracellular Ca^2+^ [54], endogenous fatty acids [67] as well as translational modifications of histones, DNA, and transcription factors which critically depend on the supply of specific mitochondrial-derived metabolites [68].

### 3.2. Mitochondrial Phenotype Shifts Associated with Commitment and Differentiation

In response to extrinsic or cell-autonomous factors, stem cells can undergo asymmetric division. In this process, one daughter cell commits to the formation of one or more types of fully differentiated cells, while the other exits the cell cycle and returns to the quiescence state, thereby contributing to renew the stem cell pool. There is now a consistent body of evidence showing that moving away from the above-mentioned *metabolic stemness* properties provokes stem cell commitment [65,68,71,72], and is required for the subsequent process of differentiation which is generally associated with major changes in the ultrastructure and molecular makeup of mitochondria [44,73,74,75].

As shown in Figure 2, activation and commitment are associated with an overall increase in cellular metabolic rates which serves in part to meet the energy requirement of proliferation. Several studies have shown that these immediate needs are met through stimulation of glycolysis, and de-repression of pyruvate oxidation in mitochondria [72,76,77,78,79,80,81]. This not only results in a burst in mitochondrial ROS production which promotes proliferation and differentiation [51,52,54,60] but also alters the concentration of multiple mitochondrial metabolites regulating signaling pathways and gene programs involved in fate determination and early differentiation including NAD^+^/NADH, AMP/ATP, TCA cycle intermediates, Acetyl-CoA and SAM/SAH [65,68,82,83,84,85,86,87,88].

Once committed to a specific lineage, progenitor cells undergo further mitochondrial remodeling in order to support the specific needs of terminally differentiated cells. For progenitor cells destined to differentiate into cardiac myocytes, skeletal muscle fibers or neurons, which have high and/or sustained energy requirements, remodeling includes an increase in mitochondrial content and structural complexity, enhanced capacity for OXPHOS, and improved antioxidant capacities to cope with the electron leaks resulting from the increased metabolic activity [44,73,74] (Figure 2). On the other hand, for cells such as HSCs, mitochondrial changes observed during terminal differentiation of progenitor cells varies considerably throughout the blood cell hierarchy. For instance mitochondria are completely eliminated during erythropoiesis to fulfill the O_2_-transport function of red blood cells [89], while in neutrophils and eosinophils, mitochondria are preserved, but fine-tuned for apoptosis and ROS production rather than for energy production [90], suggesting that highly orchestrated and lineage-specific mitochondrial remodeling programs are required for normal differentiation. Consistent with this notion, several studies have shown that preventing mitochondrial remodeling or inhibiting respiratory function severely impairs differentiation of multiple types of progenitor cells, including cardiac muscle alongside other progenitor cells [44,73,74,75].

## 4. Mitophagy in Stem Cells

Numerous studies have convincingly demonstrated that macro-autophagy plays a crucial role not only in maintenance of self-renewal capacity but also in the commitment and differentiation of stem cells [71,91]. In general, these studies have shown that pharmacological inhibition of macro-autophagy (ex: 3-MA), or genetic inactivation of core autophagy genes (e.g., *Atg3*, *Atg5*, or *Atg7*) or key regulators of mTOR activity (e.g., *Raptor*, *Lkb1*, *Tsc*, *Fbw7*) profoundly impairs the ability to maintain quiescence, prompts cellular senescence, and blocks differentiation [71,92,93,94,95,96,97,98]. These dramatic consequences are not unexpected considering that disruption of core components of this major recycling pathway leads to a general disruption of cellular proteostasis, accumulation of defective organelles (including mitochondria) and a severe impairment of nutrient sensing and nutrient supply mechanisms [99]. On the other hand, the role of specific forms of autophagy is less understood. Emerging evidence however suggest that mitophagy is likely involved in the control of stem cell fate, and proper differentiation into specific lineages.

### 4.1. Mitophagy and iPSC Reprogramming

Studies focused on reprogramming of somatic cells to induced pluripotent stem cells (iPSCs) provide support for a potentially important role of mitophagy in stem cells fate decision [78,100]. More specifically, Vasquez-Martin et al. [78] reported that reprogramming of mouse embryonic fibroblasts (MEFS) into iPSCs was delayed and less efficient in absence of PINK1. Furthermore, PINK1-deficient iPSCs colonies, which were characterized by a mixture of mature and immature mitochondria, appeared unstable, with a strong tendency to spontaneously differentiate and form heterogeneous populations of cells [78]. When tested in vivo, PINK1-deficient iPSC also showed a markedly reduced capacity for teratoma initiation, and lower levels of poorly differentiated primitive appearing stem cells consistent with an impaired ability to retain stemness properties [78]. On the other hand, pluripotency and ability to differentiate was preserved. Along the same line, reprogramming of MEFS into iPSCs through the expression of Sox2, Klf4, Pou5f1/Oct4 and Myc/c-Myc (SKPM/SKOM) was recently shown to involve an increased expression of BNIP3L/NIX and a transient upregulation of mitophagy to enable mitochondrial remodeling [100]. Furthermore, knockdown of BNIP3L/NIX blunted mitochondrial remodeling and significantly reduced reprogramming efficiency [100]. Together, these studies [78,100] provide indication that the “switching off” of the mitophagy machinery may be important for the progression of stem cell differentiation, and suggest a role for this mitochondrial quality control mechanism in maintaining stem cell identity.

### 4.2. Mitophagy in Stem Cell Maintenance

Evidence that active mitophagy is linked to the maintenance of self-renewal capacity were also obtained in studies on reconstitution of hematopoiesis [101]. Transcriptomics analysis comparing populations of HSCs with high (Tie2^+^-HSCs) vs. low (Tie2^−^-HSCs) reconstitution capacity revealed elevated expression of *Pink1*, *Parkin* and *Optineurin* and greater colocalization of mitochondrial (TOM20) and lysosomal markers (LAMP1) in Tie2^+^-HSCs, suggesting enhanced mitophagy in HSCs with high self-renewal capacity [101]. Tie2^+^-HSCs also presented a transcriptomic signature of elevated mitochondrial FAO, consistent with the above mentioned *metabolic stemness* profile [101]. Interestingly, *Pink1* expression was transcriptionally regulated by PPAR (Peroxisome Proliferator Activated Receptor), a master regulator of FAO, and PPARδ agonists not only stimulated FAO but also activated mitophagy through the recruitment of PARKIN to mitochondria [101]. Moreover, promoting FAO through PPAR enhanced self-renewal of Tie2^+^-HSCs in a PINK1/PARKIN dependent manner [101]. The effect of active mitophagy on stem cells was further demonstrated in in vivo gain of function studies where hyperactivation of PINK1/PARKIN-dependent mitophagy through HSC-specific deletion of the mitochondrial AAA-protease ATAD3, caused a significant reduction in differentiated blood cells with a concomitant expansion of the HSC pool [102]. Taken together these results support the notion that active mitophagy is part of, or helps to maintain the mitochondrial profile of stemness (Figure 2).

**Figure 3:**
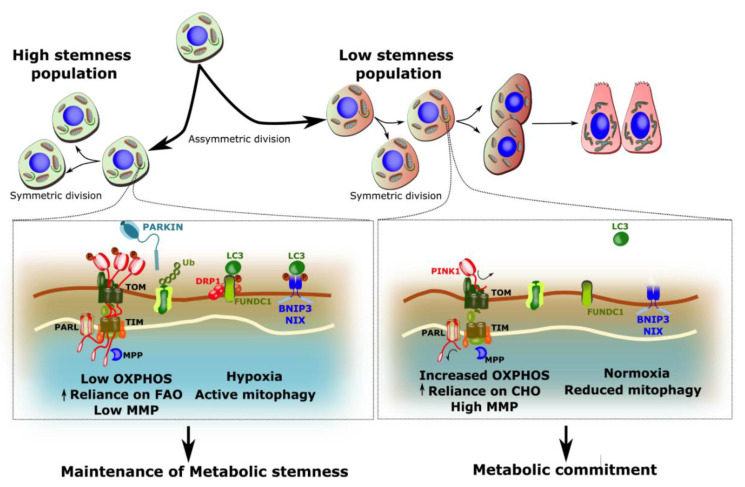
Model presenting potential mechanisms linking mitophagy to the metabolic landscape of stem cells versus committed cells. Mitochondria within stem cell populations have low rates of OXPHOS, predominantly use Fatty Acid Oxidation (FAO), and are partially depolarized (low MMP). Mechanistically, this can promote PINK1 import into mitochondria, leading to their clearance through PARKIN-mediated mitophagy. Furthermore, oxygen levels is suggested to be low (hypoxia) in specific stem cell niches such as bone marrow, which may increase the expression and recruitment of hypoxia-inducible mitophagy regulators, such as BNIP3 and FUNDC1 [24,25,106]. In this model, sustained mitophagy would enable pruning of the mitochondrial pool, and maintenance of metabolic stemness. Conversely, transition to increased OXPHOS, de-repression of pyruvate oxidation, high MMP, and higher oxygen levels (normoxia) result in the inhibition of mitophagy in order to facilitate the shifting metabolic profile committing cells. Based on this model, specific impairment of mitophagy is thus predicted to promote premature commitment and impair long-term maintenance of the stem cell pool. (Ub, ubiquitin).

An interesting study by Liang et al. provides insights on the potential mechanisms underlying active mitophagy in self-renewing HSCs [58]. The authors reported greater recruitment of PARKIN and enhanced colocalization of TOM20 and LAMP1 to mitochondria in quiescent self-renewing HSCs compared to cycling-primed HSCs [58]. Interestingly, mitochondria in quiescent self-renewing HSCs presented low MMP compared to cycling-primed HSCs [58]. Given that loss of MMP is a key event leading to the activation of mitophagy [103,104], these data suggest that partial depolarization of mitochondria could represent a mechanism to maintain mitophagy active in quiescent HSCs. In these cells, partially polarized mitochondria may be prone to accumulate uncleaved PINK1 at their surface, leading to the recruitment of PARKIN and the autophagy machinery. Although the mechanisms underlying low MMP remain uncertain, the reduced rates of OXPHOS and the use of fatty acids which are known to their uncoupling effects [105] could be involved as illustrated in Figure 3. Although this is increasingly debated, some stem cells are suggested to reside in hypoxic niches. Therefore, low PO_2_, aside from reducing rates of OXPHOS, could also promote mitophagy through FUNDC1 and/or BNIP3-NIX which are hypoxia-sensitive [24,26] (Figure 3).

Using stem-like epithelial cells, Katajisto et al. provided evidence for the importance of mitochondrial age in the maintenance of stemness [107]. By monitoring fluorescently labelled mitochondria these authors observed an asymmetric separation of mitochondria in stem-like epithelial cells (SLC), whereby older mitochondria were preferentially segregated into one of the two daughter cells [107]. Importantly, the population of cells with older mitochondria were observed to have less stem-like properties, including a limited capacity to form mammospheres (a marker of stemness in SLCs). When altering the mitochondrial quality control state of these SLCs, by knocking down *Parkin* or using the mitochondrial fission inhibitor Mdivi-1, the population of SLCs containing only young mitochondria was dramatically reduced. Furthermore, there was an associated inability to form mammospheres [107]. This has been further demonstrated in clonal selection of B and T lymphocytes where an unequal elimination of aged mitochondria was observed in self-renewing lymphocytes compared to those undergoing differentiation [108]. Together with the above-mentioned results linking the reliance on FAO, and the low metabolic state of mitochondria (i.e., low MMP, low oxygen consumption) to stemness, these data would thus suggest that mitophagy allows to limit the accumulation of old mitochondria that have lost these *metabolic stemness* features.

### 4.3. Mitophagy in Stem Cell Differentiation

Several pieces of evidence also suggest a role for mitophagy in stem cell differentiation. For instance, in the developing mouse embryo, studies have suggested a role for mitophagy in the development of retinal ganglion cells (RGCs). More specifically, it was shown that differentiation of neuroblasts into RGCs required active mitophagy in order to transiently downregulate mitochondrial content and shift cellular metabolism toward glycolysis, the latter being viewed as a key event in the differentiation of RGCs [109]. Mechanistically, this response appeared to be mediated through a hypoxia-dependent mechanism and driven by NIX-dependent mitophagy [109].

The increase of mitophagy at the onset of differentiation has also been observed in muscle differentiation [110]. This was demonstrated during the transitions from myoblast to myotube, which required mitophagic clearance of mitochondria coupled with mitochondrial biogenesis to produce a switch from glycolysis to OXPHOS [110]. Although this was initially described in cultured C2C12 myoblasts [110], further work has suggested the importance of autophagy and possibly mitophagy in primary muscle stem cells (i.e., satellite cells or SCs) function and activation [111]. In these cells, it was established that autophagic flux was induced during stem cell activation, while inhibition of autophagy suppressed the ability to increase ATP levels and delayed stem cell activation [111]. This was examined further under the context of ageing where basal rates of autophagy in SCs were found to be decreased, resulting in an impaired ability to maintain quiescence [112]. This study further showed that in aged senescent SCs, mitophagy was inhibited and ROS levels were elevated, suggesting an important role for mitochondrial quality control in preventing SC senescence [112].

Mitophagy was also found to be rapidly induced upon initiation of differentiation in Cardiac Progenitor Cells (CPCs) [106]. This response, which was mediated by the mitophagy receptors, BNIP3L/NIX and FUNDC1 rather than the PINK1/PARKIN pathway, appeared to facilitate the CPCs to undergo proper mitochondrial network reorganization during differentiation. As a result, abrogating BNIP3L- and FUNDC1-mediated mitophagy during differentiation was found to lead to sustained mitochondrial fission and formation of donut-shaped impaired mitochondria [108]. Interestingly, the same study reported that knockdown of BNIP3L/NIX and FUNDC1 had no impact on progenitor cell fate determination or reprogramming [106], which contrasts with the above mentioned results suggesting an important role of NIX in iPSC reprogramming from MEFs [100]. This apparent discrepancy could illustrate the existence of tissue/cell specificity of different mitophagy pathways, and to a degree that stem cells do not all have the same requirements for mitophagy.

Based on the available evidence, the emerging picture is that mitophagy plays a dynamic role in stem cell fate decision, lineage progression and differentiation. As illustrated in Figure 2 and Figure 3, mitophagy rates likely influence the propensity of stem cells of self-renew or commit by effecting the proportion of mitochondria displaying the typical characteristics of stemness vs. activation/commitment, which we respectively term *metabolic stemness* and *metabolic commitment* properties. Moreover, activation of mitophagy is required in several cellular lineages to support extensive structural and biochemical remodeling of mitochondria taking place during differentiation. More studies will surely clarify mechanisms and provide further insight into the stem cell or tissue specific requirements for mitophagy in ensuring an optimal balance between self-renewal and differentiation.

## 5. Mitophagy in Disease and Treatment

**Figure 4:**
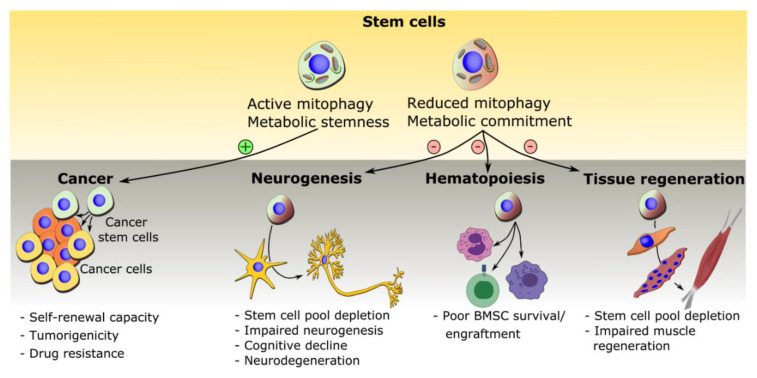
Stem cell mitophagy: implications in diseases. Emerging evidence indicate links between stem cell mitophagy and the development of disease. In the central nervous system, reduced mitophagy is neural stem cells is suggested to lead to depletion of the neural stem cell pool, which in turns leads to impaired neurogenesis [113,114,115,116,117]. Impaired mitophagy and neural progenitor cell defects are believed to be present in several conditions including mood disorders (Autism [118], Schizophrenia [119,120]), cognitive decline (Alzheimer’s) [121], and neurodegeneration (e.g., Parkinson [114,122], Huntington’s [123,124,125]. Recent work has highlighted the importance of mitophagy in enhancing survival and engraftment of bone marrow mesenchymal stem cells [45]. Defective mitophagy is stem cells is also predicted to lead to impaired tissue regeneration. For instance, in skeletal muscle, defective macro-autophagy, which also impairs mitophagy, leads to stem cell pool depletion and impaired muscle regeneration [112]. Mitophagy in cancer stem cells is suggested to contribute to drug resistance [126,127]. Furthermore, sustained mitophagy in cancer stem cells could enhance self-renewal through the maintenance of metabolic stemness, which could then contribute to tumor growth [128].

The new-found prominence of active mitochondrial mechanisms in promoting stem cell functions has led to a progressive way of thinking in terms of implications in disease settings. With the increasing documentation of mitochondrial dysfunctions in aging and a vast array of diseases, most notably degenerative diseases, one begins to think of the implications this would have on stem cell function and the maintenance of tissue homeostasis. The following question would then logically arise: would the presence of dysfunctional mitochondria within a given disease cell not inadvertently implicate potential disruptions in mitochondrial quality control checkpoints and mechanisms? If true, then not only would mitophagy be an important player in stem cell biology, but defects in this process could be present within stem cells in the context of diseases and aging. This has been observed in a number of disease states with both increased and decreased mitophagy playing a role in disease progression. Figure 4 demonstrates some of the known dysfunctions observed with changes to mitophagy and here, we review recent advances connecting mitophagy in disease conditions and therapeutic interventions.

### 5.1. Mitophagy in Cancer Stem Cells

The role of mitophagy in cancer is a complex relationship. Changes in expression of mitophagy pathways both up and downregulation can result in worse prognosis for patients [129]. The discovery of cancer stem cells (CSCs), a unique subpopulation within tumors responsible for self-renewal, has led to the investigation of mitophagy in these subtypes [130,131,132]. Different CSC subtypes have unique mitochondrial characteristics, by way of their dynamics, metabolism and ROS levels. For example, some models of CSCs, such as nasopharyngeal and lung CSCs, contain mitochondria in an immature state reminiscent of those observed in embryonic stem cells and iPSCs [133,134], while pancreatic CSCs, gliomaspheres, have been reported to rely on mitochondrial OXPHOS for energy production [135]. Thus with differing states of mitochondria among CSCs subtypes and considerations for heterogeneity of tumors [135], there appears to be a model specific difference in the reliance on mitophagy in CSCs [136]. Nonetheless, PINK1-dependent mitophagy appears critical in the maintenance of hepatic CSCs by sequestering p53 and upregulating the NANOG pathway [128]. In addition, the overexpression of the mitochondrial DRP1 receptor FIS-1 has been described in AML stem cells (LSCs), causing increased mitophagy and altered mitochondrial ultrastructure and ROS production [128]. Interestingly, depletion of FIS-1 using targeted siRNA led to a significant reduction in LSC self-renewing capacity. While the study mainly focuses upstream of mitophagy on FIS-1 which plays an important role in mitochondrial dynamics, the authors use valinomycin to stress the FIS-1 silenced cells mitochondria, demonstrating a reduction in mitophagy and a decrease in PINK1 expression compared to controls. The loss of colony forming units is also observed in this study with just siRNA silencing of PINK1 in the LSCs. The authors then speculate about the importance of mitophagy for stem cell and progenitor potential of AML as a downstream effect of dynamics [137].

Mitophagy in some CSCs has been determined to be a major contributor to drug resistance in some tumors. In colorectal CSCs exposed to the chemotherapeutic doxorubicin, it was determined that the enhanced mitophagy, which was determined by an increased TOMM20/LC3B colocalization was responsible for the drug resistance observed in CSCs. While the expression of PINK1 and PARKIN had not significantly changed with doxorubicin, NIX was significantly upregulated. Inhibition of general autophagy by ATG7 did not improve sensitivity to chemotherapy, but when NIX was silenced, sensitivity to the drug was improved [126]. Although the PINK1/Parkin pathway had no effect on doxorubicin treatment, it has been used to improve drug resistance in colorectal CSCs. Mefloquine inhibits the RAB5/7 pathway, preventing the formation of endosomes, this leads to the suppression of PINK1 and Parkin mediated mitophagy: this was detected by immunostaining in cells treated with mefloquine [127] This treatment significantly reduces the number of CSCs as did the siRNA knockdown of RAB5/7 and PINK1. This lack of mitophagy resulted in increased sensitivity to traditional chemotherapeutic agents improving tumor reduction in three xenograft tumor mouse models.

### 5.2. Mitophagy in Neural Stem Cell Depletion and Impaired Neurogenesis

In neural stem cell populations, the importance of mitochondria is well known in controlling metabolism, ROS signalling and stem cell fate decisions [138]. Diseases of neurogenic degeneration such as Parkinson’s and Alzheimer’s disease are a large focus of study in this area. New developments in this area have shown impaired neurogenesis in these neurodegenerative disorders. In Alzheimer’s disease, mutations in the presenilin 1 (*Ps1*) gene reduces stem cell proliferation, survival and neuronal differentiation in knock-in mice [113]. Importantly, enhancing mitophagy using Bexarotene in human iPSCs, generated with this mutation (PS1 M146L mutation), promoted improvements in the function of these cells [121]. Martín-Maestro et al. suggest bexarotene treatment enhanced autophagy; this general increase in macro-autophagy although not technically a specific enhancer of mitophagy restored the mitochondrial network and increased TOM20-LAMP1 co-localisation, suggesting an increase in mitophagy as well [121]. This was also demonstrated by increased ubiquitin phosphorylation at Ser65, which activates Parkin and increases its colocalization with the mitochondria. Thus, one can speculate that enhanced mitophagy, alongside autophagy, with bexarotene treatment could be responsible for the rescue effects observed [121].

Evidence from animal models also shows that disruptions in neurogenesis in Parkinson’s disease [114]. The PINK1 knockout mouse has impaired neuronal maturation in the dentate gyrus of the adult hippocampus [114]. Here, the doublecortin-positive newborn neurons had aberrant dendritic morphology and their maturation was impaired. Though, there was no observed effect on the number of neural stem cells (NSCs) in vivo, cultured adult hippocampal PINK1-deficient NSCs displayed reduced growth rates, increased apoptosis and mitochondrial dysfunction, as observed by loss of membrane potential and increased reliance on glycolysis [114]. These factors certainly suggest a role for abnormal mitophagy in the control of NSCs and neurogenesis in Parkinson’s disease. Defects in mitophagy have also been observed in human neuroepithelial stem cells (NESCs) carrying LRRK2-G2019S, a causative mutation in Parkinson’s disease [122]. NESCs displayed a deficiency in mitophagy, which were linked to decreased mitochondrial function and cell death [122].

Huntington’s Disease is another neurodegenerative disease which also encompasses a neurodevelopmental aspect to the disease progression [115]. As such, deficits in brain growth were observed in children at risk of developing Huntington’s disease [139] and numerous animal studies have suggested the importance of huntingtin (HTT) in brain development [115,140,141,142]. Of particular interest here, is the reported involvement of impaired autophagy in this disease, with a specific effect of mutant HTT in impairing autophagasome trafficking [124]. One can envision how defects in autophagasome trafficking would have a severe consequence on mitophagy as well. More importantly, a recent report has discovered that HTT is involved in the interaction between damaged mitochondria and the nascent autophagosome and recruiting mitophagy receptors [143]. Of particular interest here, the knock-in Hdh-Q111 mouse model of HD has aberrant expression of neurogenesis markers and abnormal cell cycle progression [142]. When transferred to a culture model these NSCs from this animal model demonstrated limited differentiation capacity, reduced proliferative potential and enhanced late-stage self-renewal [142]. Interestingly the knock in NSCs also demonstrated upregulation of the NANOG pathway which has been implicated to be altered by PINK1-dependant mitophagy in hepatic CSCs [128] and is a well-known marker of pluripotency and enhancing self-renewal [144]. These factors demonstrate a possible role for mitophagy in development. More complete evidence has been demonstrated in terminally differentiated neurons from iPSCs carrying HD mutations: GABA MS-like neurons were differentiated from iPSCs derived from human patients with HD, these neurons had increased evidence of mitophagy. This appears to be an observation made from electron microscope images without quantification [145]. Evidence for defective degradation of mitochondria has been observed in mice with inefficient degradation of engulfed mitochondria described in primary mouse neurons [123]. Further to this PINK1 has been described as downregulated in oligodendrocytes and neurons of the caudate from HD patients [124,125]. This demonstrates that mitophagy could play a role in the differentiated tissue effected by HD, this added to the changes in early neurogenesis which have also been described in PD and Alzheimer’s could suggest a role for mitophagy in these stem cells which could possibly be an avenue for further study in HD.

Study of the development of Autism Spectrum Disorder (ASD) found a possible link to dysfunctional mitophagy and neuronal stem cell dysfunction. Deficiency in the Wdfy3 gene in mice regulates neural progenitor divisions, with increased proliferation of the early progenitor cells and reduced number of intermediate progenitors. Neural migration was also severely impaired in the developing brain and the cell cycle length was shortened by approximately 30% [116]. The Wdfy gene was then subsequently determined to be heavily involved in mitophagy and mitochondrial quality control [118]. Interestingly, changes in the early progenitor cells share similarities to other possible mitophagy related complications, suggesting this may warrant future investigation in the development of ASD.

Development of schizophrenia may also be affected by impaired mitophagy. Disrupted-in-schizophrenia-1 (DISC1) is a protein that has been studied extensively for its role in numerous physiological disorders [146]. Loss of DISC1 is implicated in impaired neurogenesis and proliferation of embryonic and adult neural progenitor cells through the GSK3β/β-catenin pathway [147]. It has also been shown that loss of DISC1 inhibits dendrite development during neuronal maturation from newborn dentate granule neurons [117]. Importantly here, DISC1 has recently been described as a mitophagy receptor as it can localize to the mitochondrial membrane and bind directly to LC3 [119,120]. Thus, modifications to DISC1 demonstrate changes to neuronal development while also showing possible links to mitophagy.

### 5.3. Mitophagy in Bone Marrow Mesenchymal Stem Cells and Bone Diseases

The upregulation of mitophagy in Bone marrow mesenchymal stem cells (BMSCs) prior to transplantation has been shown to improve the survival rate of BMSCs and subsequently its reparative ability [45]. BMSCs transplantation is considered an effective method for the treatment of bone diseases including osteonecrosis of the femoral head (ONFH) but due to their multi-potential capacities to differentiate into multiple lineages, there remain major limitations to due to the very low survival rate of the transplanted BMSCs [45,148]. Poor engraftment and short term survival of transplanted MSCs have been linked to several factors such as increased ROS levels, stress-induced apoptosis and senescence in the damaged area [45]. Many studies have reported that high levels of ROS can be detrimental for the survival and maintenance of stem cells including MSCs [149,150,151]. Zhang et al. further explored the link between ROS-induced accumulation of damaged mitochondria and the low survival rate of transplanted BMSCs. They revealed ROS-induced apoptosis, an increase of the senescence marker, β-gal and higher expression of the aging-related markers, P21 and P16. Furthermore, a recent study also showed that mitophagy protected MSCs against senescence during ex vivo expansion for clinical applications [152]. Increase in mitophagy has also been shown to be beneficial for BMSCs by restricting inflammasome activation therefore supporting its protective role [153]. This led to the speculation that mitophagy as a mitochondrial quality control mechanism could clear these dysfunctional and damaged mitochondria and thus protect the transplanted BMSCs. Following transplantation of BMSCs, upregulation of Parkin and downregulation of P53 completely repaired the defected area in the osteonecrotic mice [45] thus suggesting that induction of mitophagy could be an effective treatment for osteonecrosis and other bone diseases.

### 5.4. Mitophagy in Ageing

Mitochondria play an important role in ageing, whether through mtDNA mutations, inflammation, redox dysregulation or quality control [154]. The role of mitophagy in stem cells during ageing is a developing topic with a number of different studies suggesting a possible significant role for mitophagy in this process. In muscle, García-Prat et al. have reported that basal autophagy is required for the maintenance of quiescence satellite cells. Addition of rapamycin to geriatric satellite cells, used to increase autophagy, was able to restore mitochondrial content and diminish ROS levels in these aged stem cells [112], suggesting mitophagy may play a role in this process. Similar processes have also been observed in HSCs with aged stem cells showing a higher proportion of metabolically active mitochondria with larger more fused networks [98]. The old stem cells were more reliant on mitochondria and OXPHOS for metabolism determined by bioenergetic flux analysis (Seahorse Technologies), which the authors demonstrated to also be the case in young autophagy deficient HSCs with Atg12 mutations. The authors suggest that autophagy and in turn mitophagy may be important for the removal of active mitochondria in young stem cells and is hindered in aged HSCs [98]. Similarly, depletion of PINK1 and Parkin in intestinal stem cells (ISCs) from *Drosophila* results in increased senescence [155]. SiRNA-mediated knockdown or PINK and Parkin resulted in changes within mitochondria ultrastructure, observed by 3D electron tomographic reconstructions, showing mitochondria were ultra-condensed with enlarged intra-cristae space and a reduced matrix. As was observed in aged HSCs, disruption of mitophagy led to a higher reliance on mitochondrial OXPHOS [98,155]. In addition, reduction of PINK1/Parkin in ISCs caused a severe reduction in proliferation and increased expression of senescence markers β-galactosidase (SA–β-gal) and HP-1. There was also observed increases in ROS in aged control stem cells which was also observed in young and aged PINK1 and Parkin knockdowns [155]. Given the relevance of senescence in ageing, these observations draw a close relationship between ageing and mitophagy in stem cells. This idea is further strengthened by Zhang et al., where upregulation of Parkin in BMSCs increased mitophagy and promotes the down-regulation of age- and senescence-related markers [45].

In the premature ageing disease Werner syndrome, impaired mitophagy and depletion of NAD+ have recently been implicated in disease progression [156]. Repletion of NAD+ in a *C. elegans* model of Werner syndrome improved the number and proliferative potency of mitotic cells, improving the health and lifespan of the worms, indicating an improvement in stem cell function [156]. This was also observed in an ISC drosophila disease model, where NAD+ supplementation precursors improved the lifespan and proliferative potential of ISCs. Importantly these NAD+ mediated effects were reliant on the NIX homologue pathway DCT-1 [156]. The involvement of mitophagy was then further solidified using the specific mitophagy inducer Urolithin A, which rendered similar effects as NAD+ supplementation and was shown to improve lifespan [156].

### 5.5. Therapeutic Interventions

While mitophagy appears to be playing a role in the development of a number of diseases cited in this review, targeting this pathway for clinical interventions has proved a difficult task. The main issue being the lack of pharmacological agents that can specifically target the mitophagy pathway, as opposed to the availability of agents that can be used to manipulate macro-autophagy. For example, as seen in mouse models of muscle ageing induction of macro-autophagy with rapamycin was able to stimulate improvements [112]. As was the case for PS1 M146L mutant mice treated with bexarotene to increase overall autophagy [121]. While this provides a first step in the target of mitophagy there is divergence between the two pathways which are not always linked. There are also some concerns with the alteration of bulk autophagy as seen in rapamycin studies and its role in the development of diabetes/hyperglycemia [157].

Pharmacological agents specifically altering the mitophagy process are few and far between. The P62-mediated mitophagy inducer (PMI), has been determined to increase mitophagy without recruiting PARKIN or collapsing the membrane potential and maintains bio-energetic competence of the network [158]. This also appears to stabilize NRF2 an important antioxidant signaling pathway. While PMI has a promising mechanism of action studies in humans have not been published, one such drug which has already undergone human testing is the mitophagy inducer Urolithin A [159]. In this study elderly individuals were treated over a four-week period and an upregulation of plasma acylcarnitines and skeletal muscle mitochondrial gene expression was observed., suggesting an improvement in mitochondrial and skeletal muscle health [159]. However, the mechanism of action by which Urolithin can improve mitophagy is not entirely clear. Furthermore, whether Urolithin is specifically inducing mitophagy, rather than macro-autophagy in general has not been validated [160,161]. Recently, efforts have been put towards small-molecule screens to identify mitophagy modulators. As a result of a small-molecule screen looking for potentiators of Parkin to specifically target mitophagy, Rho-associated protein kinase (ROCK) inhibitors have become the latest target for the possible pharmacological manipulation of mitophagy [162]. The authors of the study demonstrate an increase in mitochondrial hexokinase 2 (HK2) and the subsequent recruitment of Parkin to mitochondria [163]. While this study is promising, ROCK inhibition is known to effect multiple other pathways: cytoskeleton organization, cell adhesion, proliferation, apoptosis etc. [162]. Thus, the specificity of these modulators and may be an issue here as well. Overall, it appears that although mitophagy can be modified by certain drugs, direct therapeutic interventions are currently limited and specificity appears to be an issue using these drugs to study mitophagy. Importantly, future efforts in identifying specific modulators of mitophagy will prove to be extremely beneficial.

## 6. Conclusions

Mitochondria play a valuable role in the maintenance of stem cells through the control of the metabolism, ROS production, calcium buffering, and gene expression. With the increased study and interest in macro-autophagy and its role in stem cells, further dissection of these pathways has led to the idea of mitophagy having a degree of control in these pathways. This has been demonstrated in a number of systems where mitophagy pathways have been manipulated in stem cells and have led to changes in stem cell self-renewal and differentiation capacity. Moving into disease models, it is becoming apparent that changes in mitophagy are more prominent than the classically studied effects in Parkinson’s disease. Modification of mitophagy could possibly become a treatment options or at least may be considered as part of a strategy for improving patient outcomes in complex diseases, such as drug resistance in cancer, BMSC engraftment or in ageing. The use of mitophagy modifiers have begun to show positive results, shedding some hope on potential ways to improve patient lives. Though the majority of studies have focused on the PINK1/PARKIN or NIX pathways, mitophagy involves multiple pathways of which certain aspects may be overlooked due to a degree of compensation. Thus, additional studies further dissecting different aspects of mitophagy will provide a better understanding of the role of this mitochondrial quality control mechanism in stem cell biology both in the context of development or disease settings.
